# Engineering exosomes for targeted drug delivery

**DOI:** 10.7150/thno.52570

**Published:** 2021-01-01

**Authors:** Yujie Liang, Li Duan, Jianping Lu, Jiang Xia

**Affiliations:** 1 Department of Child and Adolescent Psychiatry, Shenzhen Kangning Hospital, Shenzhen Mental Health Center, Shenzhen Key Laboratory for Psychological Healthcare & Shenzhen Institute of Mental Health, Shenzhen, 518020, China; 2 Department of Chemistry, The Chinese University of Hong Kong, Shatin, Hong Kong SAR, China; 3 Department of Orthopedics, Shenzhen Second People's Hospital (First Affiliated Hospital of Shenzhen University, Health Science Center), Shenzhen 518035, China

**Keywords:** Exosomes, targeted delivery, chemical modification, genetic engineering, exosomal membrane proteins

## Abstract

Exosomes are cell-derived nanovesicles that are involved in the intercellular transportation of materials. Therapeutics, such as small molecules or nucleic acid drugs, can be incorporated into exosomes and then delivered to specific types of cells or tissues to realize targeted drug delivery. Targeted delivery increases the local concentration of therapeutics and minimizes side effects. Here, we present a detailed review of exosomes engineering through genetic and chemical methods for targeted drug delivery. Although still in its infancy, exosome-mediated drug delivery boasts low toxicity, low immunogenicity, and high engineerability, and holds promise for cell-free therapies for a wide range of diseases.

## Introduction

In 1981, Trams et al. discovered a group of vesicle-like structures with diameters 40-1000 nm smaller than those of multivesicular bodies by transmission electron microscopy 1. Later, Johnstone et al. identified a vesicle-like structure in the process of reticulocyte maturation, and isolated these membrane-bound vesicles from sheep reticulocytes by ultracentrifugation at 100,000 ×*g* for 90 min. For the first time, these vesicle-like structures were named exosomes 2, 3. However, at that time, the discovery of exosomes did not receive much attention as these vesicles were thought to be merely waste products from maturing red blood cells. These vesicles were only recently characterized to be membrane-bound extracellular vesicles released by exocytosis after fusion of cell membrane with intracellular multivesicular bodies (MVBs) 4, 5. Exosomes are now widely found in all bodily fluids and tissues, including blood 6, urine 7, breast milk 8, amniotic/synovial/ascites fluid 9, saliva 10, and adipose tissue 11. As more and more types of extracellular vesicles have been discovered, the definition of exosome has continuously evolved 12-14.

Exosomes mediate intercellular communications. Through exosomes, donor cells can transfer exogenous substances, such as proteins, mRNAs, microRNAs (miRNAs), and lipids, to recipient cells. Consequently, these naturally-equipped nanocarriers have been used for drug delivery 15. Compared with synthetic drug carriers, exosomes isolated from a patient's own cells have higher biocompatibility and lower toxicity 16. Exosomes can also penetrate into tissues, diffuse to the blood, and even cross the blood-brain barrier (BBB) 17. Exosome-mediated delivery bypasses the P-glycoprotein drug efflux system, which can reduce drug resistance 18. Exosomes are also highly engineerable. Engineering of exosomal surface proteins confers cell and tissue specificity. Although the use of exosomes as drug delivery vehicles is the topic of many review articles 19-23, here we focus on the engineering of exosomes for targeted drug delivery. In this review, we will introduce the characteristics of exosomes as drug delivery vehicles, summarize strategies to modify the exosome membrane, illustrate applications of exosome-mediated targeted drug delivery in various disease models, and highlight the key translational challenges and opportunities.

## Biogenesis, secretion, and cellular entry

**Figure 1:**
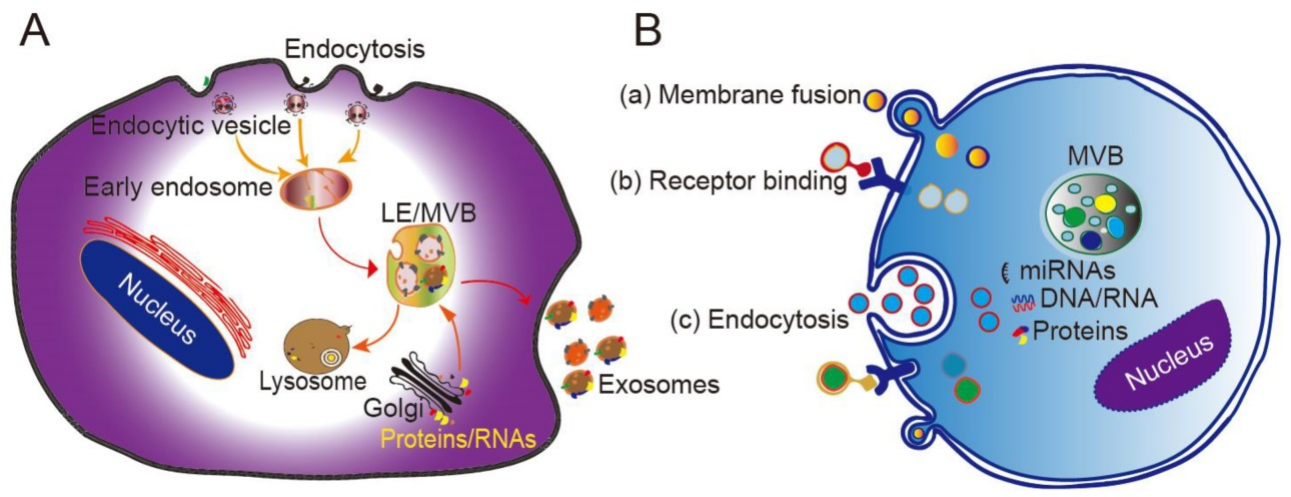
** Biogenesis, secretion, and cellular entry of exosomes.** (A) Exosomes develop within MVBs and are released by fusion with the plasma membrane. (B) Exosomes enter cells through three main pathways: (a) membrane fusion with the target cell, (b) internalization driven by receptor-ligand interactions, and (c) endocytosis. LE, late endosome; MVB, multivesicular body. Adapted with permission from 24, copyright 2014 Elsevier.

Originating from the endocytic pathway, exosomes are released from MVBs, which contain multiple vesicles generated through exocytic fusion with the cell membrane. Exosome generation includes four steps: budding, invagination, MVB formation, and secretion. As a form of budding, invagination of the cell membrane forms clathrin-coated vesicles, and clathrin vesicles enter the cytoplasm to form early endosomes. Proteins, lipids, and nucleic acids are specifically sorted and encapsulated to form multiple intraluminal vesicles (ILVs), which are the precursors of exosomes. After late endocytosis, multiple ILVs further develop into late endosomes (LEs). Then, a proportion of the LEs enters the lysosomal pathway, while the others fuse with the cell membrane, ultimately releasing multiple vesicle structures into the extracellular matrix in the form of exosomes (**Figure 1A**) 24.

Different mechanisms have been proposed to explain the formation of exosomes, with most of them acting through the endosomal sorting complex required for transport (ESCRT). The ESCRT system contains four protein complexes: ESCRT-0, ESCRT-1, ESCRT-2, and ESCRT-3 together with auxiliary proteins such as VTA⁃1, ALIX, and VPS4. These proteins act synergistically in the stepwise formation of MVBs 25. The intraluminal vesicles wrapped in MVBs are delivered to lysosomes to be degraded, or secreted directly into the extracellular environment in the form of extracellular vesicles. A recent study found that purified exosomes were enriched with ceramide, and that inhibition of the expression of sphingomyelinase reduces the extracellular secretion ability of exosomes, consistent with the mechanism of ceramide-mediated membrane budding 26. Other studies have confirmed that lipids, tetraspanins, and/or heat shock proteins are involved in the generation of ILVs and MVB sorting. For example, CD63 sorts melanoma-related proteins into human ILVs in the absence of ESCRT and ceramide 27. Furthermore, CD81 independently sorts a series of ligands into exosomes. These pathways are collectively referred to as ESCRT-independent mechanisms. Secretion of exosomes into the extracellular matrix is mainly driven by the fusion of MVB and cell membrane, and depends on the auxiliary role of several G protein families and the SNARE complex. The SNARE protein mediates the translocation and fusion of exosomes with lysosomes or cell membranes. In addition, the MVB anchoring mechanism (which is based on the substrates of the GTPases RAB7A, RAB7B, RAB35, and RALA) also affects the release of exosomes into extracellular fluid 28, 29.

The mechanism by which exosomes enter a recipient cell can involve multiple pathways at the same time. Exosomes can directly fuse with the plasma membrane, and can also be taken up through phagocytosis, micropinocytosis, and endocytosis mediated by lipid raft, caveolin, or clathrin (**Figure 1B**) 28, 29. Theoretically, exosomes can be delivered to any cell type. However, some studies have shown that exosomes can be internalized in a highly cell type-specific manner that depends on recognition of exosomal surface molecules by the cell or tissue. For example, CXCR4/SDF-1α interaction was shown to mediate the selective transfer of endothelial colony-forming cell (ECFC)-derived exosomes to the kidney, and most lymph node stroma-derived EVs containing TSPAN8-integrin alpha-4 complexes were found to be selectively taken up by endothelial cells or pancreatic cells, and internalized to a lesser extent by parental lymph node stromal cells 30, 31. Therefore, different types of exosomes may contain protein signals that serve as ligands of other cells, and these receptor-ligand interactions can be leveraged for targeted exosome delivery 32. This natural mechanism then inspires the engineering of exosomal surface proteins for targeted drug delivery.

## Cargo packaging

**Figure 2:**
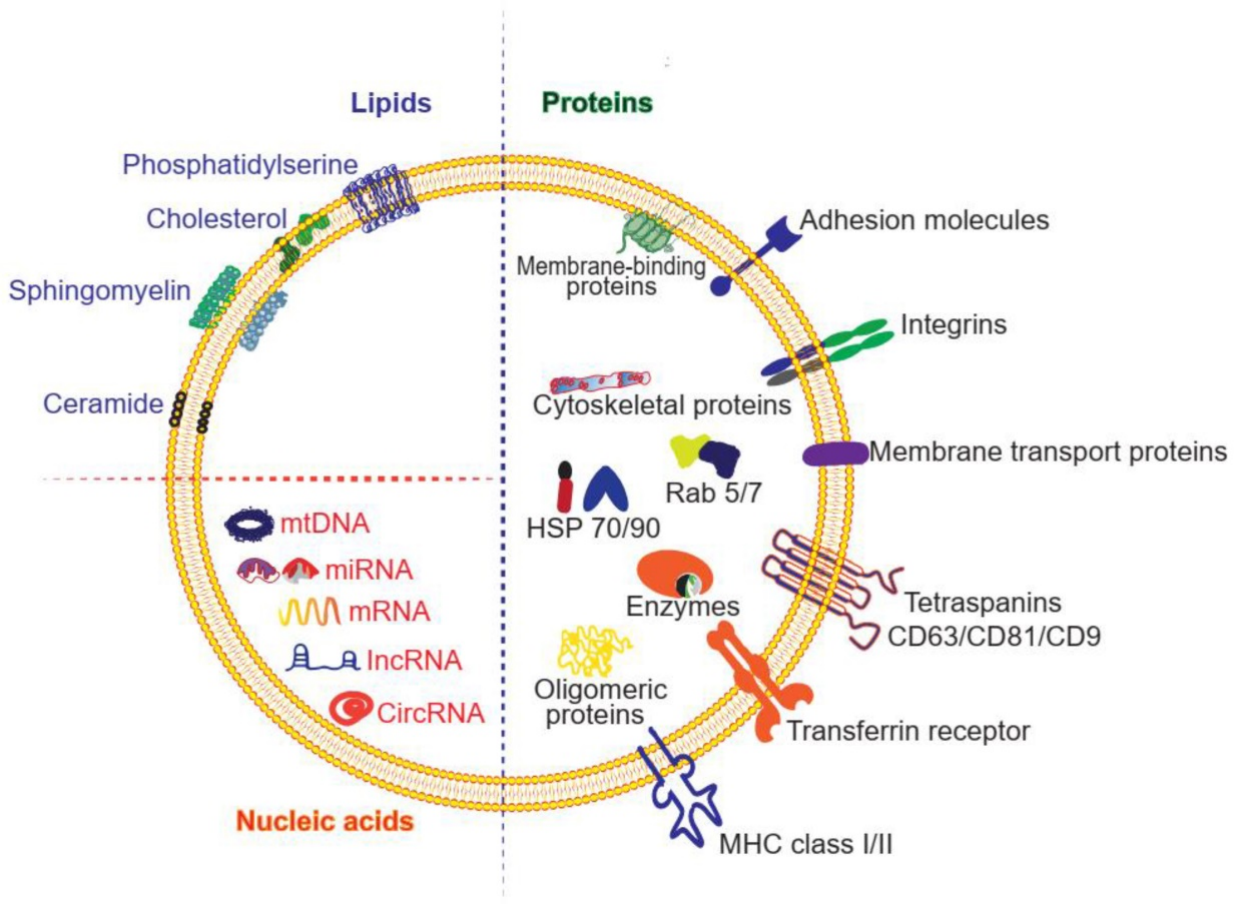
** Structure and composition of exosomes.** The molecules found in exosomes are categorized as proteins, lipids and nucleic acids. Proteins are further divided as membrane-bound proteins and soluble proteins inside the vesicles.

Exosomes can transfer various types of cargoes, including DNAs, RNAs, lipids, metabolites, and proteins (**Figure 2** and **Table 1**). To date, the ExoCarta exosome database (http://www.exocarta.org) has collected 9769 proteins, 3408 mRNAs, 2838 miRNAs, and 1116 lipids that have been identified in exosomes from different types of cells and from multiple organisms. Although the cell source determines the kinds of proteins secreted by exosomes, 80% of the proteins in exosomes are highly conserved among different cells. Some are used as biomarkers of exosomes, including ALIX, TSG101, heat shock proteins, and the tetraspanins CD63, CD9, and CD81. These transmembrane proteins account for the targeting and selective entry of exosomes to recipient cells 33. The majority of the proteins packaged in exosomes are related to the process of MVB formation, including Rab and annexin (**Table 1**). Nucleic acids are abundant in exosomes, including mRNAs, miRNAs, mitochondrial DNA and piRNAs, lncRNAs, ribosomal RNAs, snRNAs, and tRNAs. Although the nucleic acids encapsulated in exosomes are degraded fragments of approximately 200 bp, they can still impact protein synthesis inside the recipient cell. Lipids in the membrane of exosomes include cholesterol (chol), phospholipids, phosphatidylethanolamines, polyglycerols, and diglycerides. Notably, exosomes have different lipid composition, distribution, and content than the cytoplasmic membrane. The lipid molecules are involved not only in maintaining the morphology of exosomes, such as their stability and structural rigidity, but also in many biological processes. For example, they can serve as signaling mediators by interacting with prostaglandin and phospholipase C and D 34.

Loading cargoes into exosomes requires bypassing the barrier that is the exosome membrane. Synthetic carriers such as liposomes can be loaded with drugs during their synthesis. Similarly, adding exogenous drugs to donor cells preloads exosomes in situ. Co-expressing protein cargoes inside donor cells can also guide them into exosomes by designed protein-protein interactions. Preloading strategies, however, are often not an option for many types of cargoes, and so purified exosomes need to be loaded in vitro. The strategies to encapsulate exogenous molecules into exosomes can be categorized as active or passive. Electroporation is a well-utilized method for passive packaging 35. The process forms temporary pores in the exosome membrane by an electric field, through which the drugs diffuse, and then the integrity of the exosomal membrane is restored. Electroporation has been used to encapsulate all kinds of cargoes, including proteins and mRNAs. For biopharmaceuticals with suboptimal properties, loading into exosomes improves their in vivo stability, blood circulation, and cell targeting efficiency. Examples of therapeutic cargoes that have been delivered by exosomes are listed in **Table 2** and** Table 3**.

## Surface engineering

**Figure 3:**
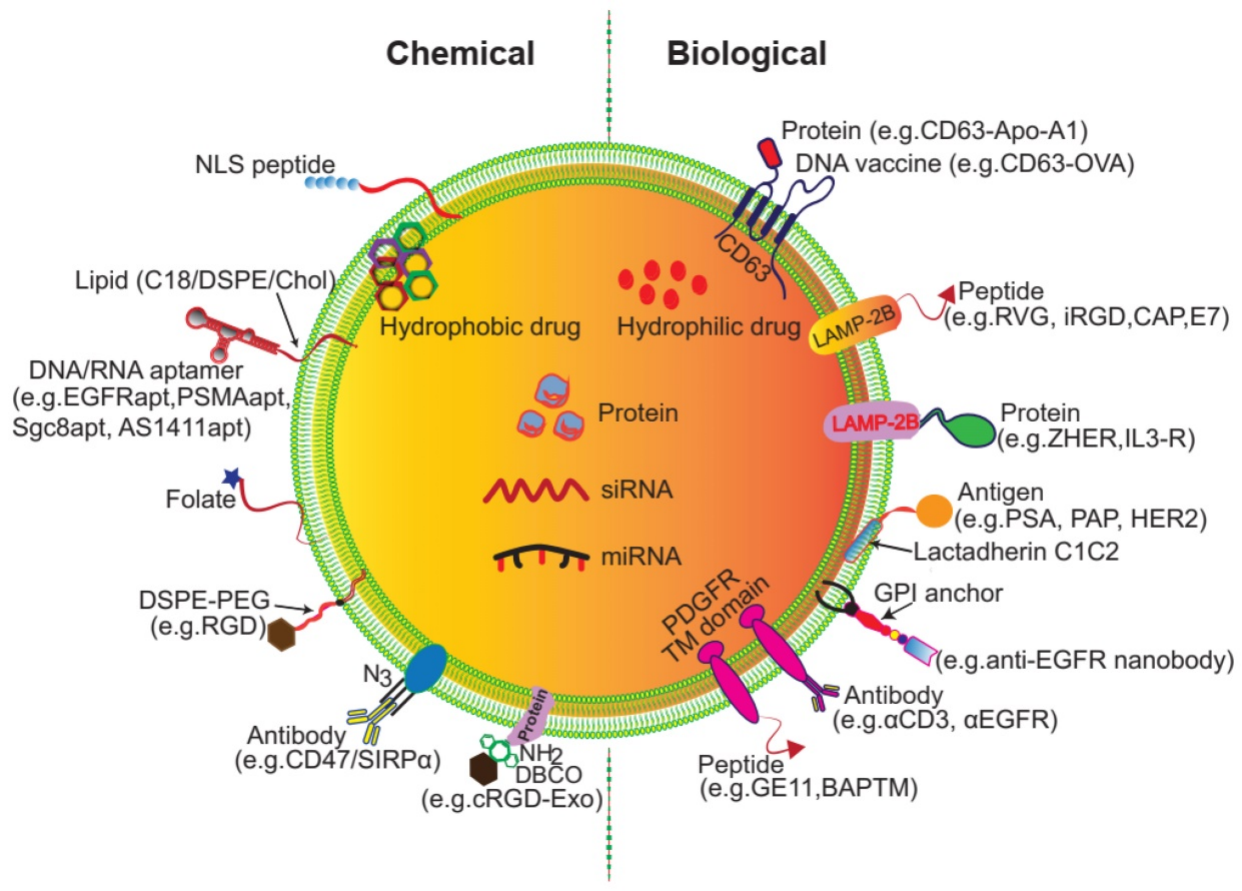
** Surface engineering of exosomes via genetic/biological manipulation or chemical modification.** Chemical modifications install different moieties, such as peptides, proteins lipids, aptamers, small molecules, polymers via chemical reactions of the lipids or membrane-bound proteins or lipid-lipid interactions. Biological manipulations introduce targeting motifs such as peptides and proteins via genetic fusion of the membrane bound proteins.

Despite being vehicles of natural origin, exosomes can be conveniently surface modified. One obvious goal of surface engineering is to confer cell type targeting specificity. Modification strategies include genetic engineering and chemical modification (**Figure 3**) 35, 42. Genetic engineering fuses the gene sequence of a guiding protein or polypeptide with that of a selected exosomal membrane protein. This approach is effective for surface display of peptides and proteins; however, it is limited to targeting motifs that are genetically encodable. Chemical modification allows a wide range of ligands, both natural and synthetic, to be displayed via conjugation reactions or lipid assembly. Conjugation reactions can covalently and stably modify exosomal surface proteins, but the complexity of the exosome surface may reduce the efficiency of the reaction, and the reaction often lacks control of site specificity. Covalent modification may also jeopardize the structure and function of the vehicle. Lipids or amphipathic molecules can also be inserted into the lipid bilayer of exosomes, allowing their hydrophilic parts to be displayed on the exterior. This method, driven by lipid self-assembly, may also increase the toxicity of exosomes. The details of these methods are elucidated below.

### Genetic engineering

Genetic engineering of exosomes is a convenient method for imparting exosomes with new properties (**Table 1**). First, ligands or homing peptides are fused with transmembrane proteins that are expressed on the surface of exosomes. Subsequently, donor cells transfected with plasmids encoding the fusion proteins secrete engineered exosomes bearing targeting ligands on their surface. Examples of genetically engineered exosomes as drug delivery systems are summarized in **Table 2**.

**Figure 4:**
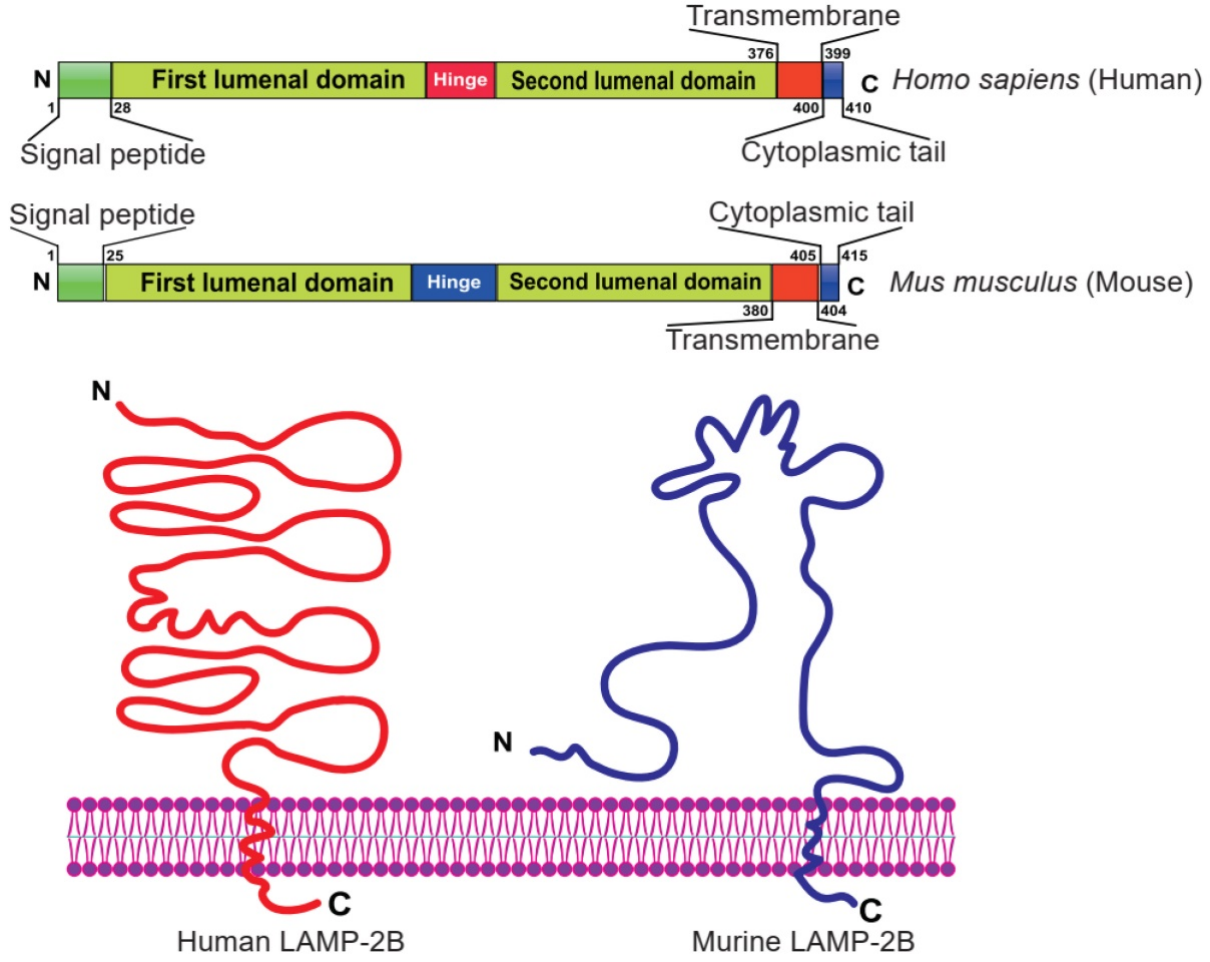
** Domain organization of human and mouse LAMP-2B.** The N-terminal portion extends into the extracellular space. Thus, ligands inserted in the N‐terminus of LAMP-2B are displayed on exosomal membranes. Adapted with permission from 45, copyright 2012 BMC, and 46, copyright 2017 Springer Nature.

Currently, LAMP-2B is the most widely used exosomal surface protein to display a targeting motif. LAMP-2B, a member of the lysosome-associated membrane protein (LAMP) family, predominantly localizes to lysosomes and endosomes, with a smaller fraction circulating to the cell surface. It has been reported that LAMP-2B protein is abundantly expressed on dendritic cell-derived exosomes. Researchers proved that the N-terminus of LAMP-2B is displayed on the surface of exosomes and can be appended with targeting sequences 35, 43. Although the full structure of LAMP-2B has not yet been revealed, the structure of the distal membrane subdomain of mouse lysosome-associated membrane protein 2 (LAMP-2) has been elucidated 44, 45. Human LAMP-2B comprises a 29 amino acid signal peptide, a large N-terminal extramembrane domain, and a C-terminal transmembrane region followed by a very short cytoplasmic tail (**Figure 4**) 46. Thus, the targeting peptide can be fused to the extracellular domain of LAMP-2B at the N-terminus.

**Figure 5:**
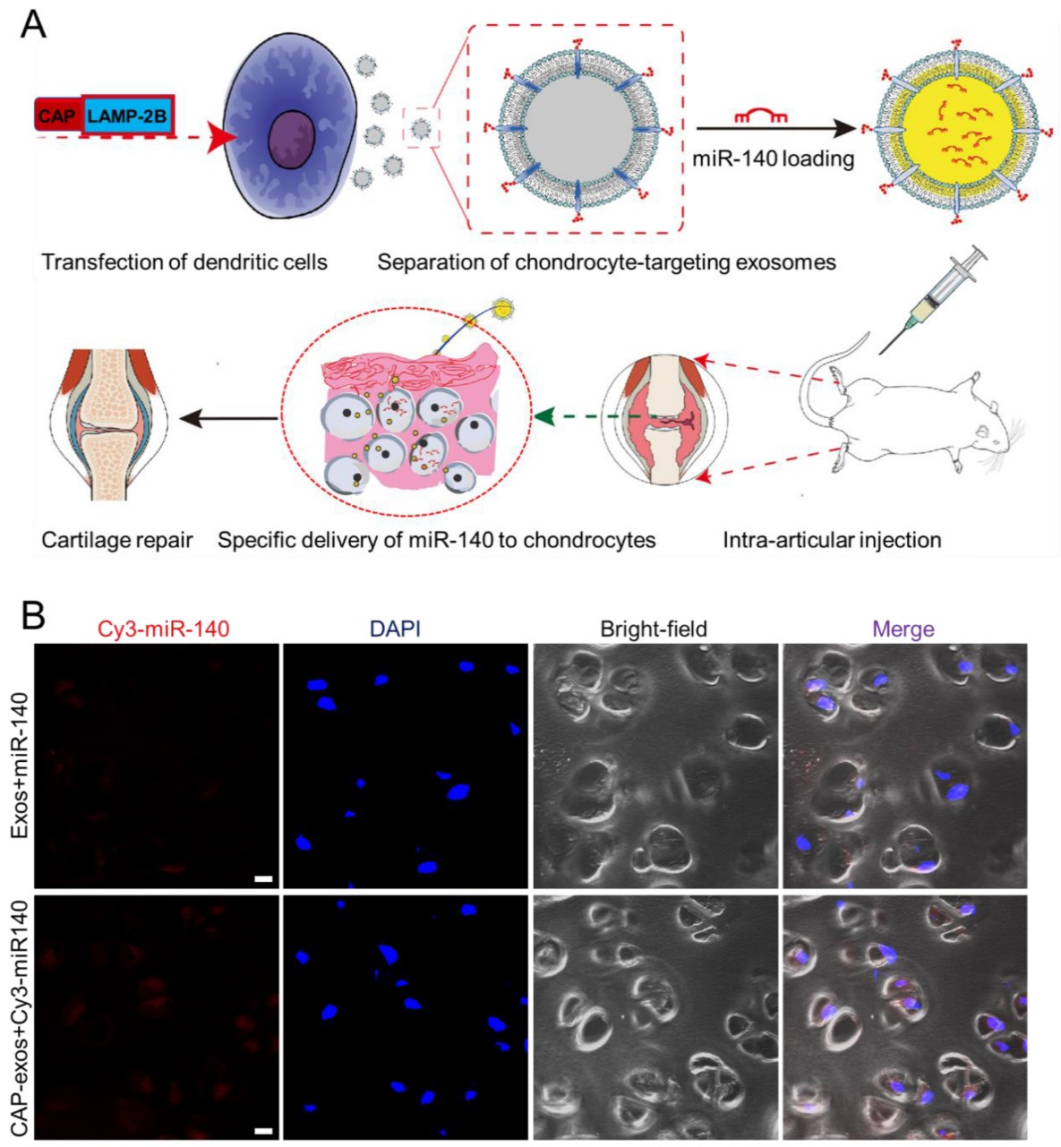
** Genetic engineering of exosomes for targeted delivery of miRNA to chondrocytes.** (A) A chondrocyte-affinity peptide (CAP, DWRVIIPPRPSA) was genetically fused with LAMP-2B and transfected into dendritic cells to produce chondrocyte-targeting exosomes. The exosomes were loaded with miR-140 and administered to a rat model of OA, where they induced cartilage repair. (B) Representative confocal fluorescence images showing uptake of Cy3-labeled miR-140 (red) from unmodified exosomes (top) or CAP-displaying exosomes (bottom) by chondrocytes embedded in cartilage tissue at 24 h after intra-articular injection. Scale bar, 20 μm. Reproduced with permission from 52, copyright 2020 American Chemical Society.

Cell-specific binding peptides targeting specific organs or tissues can be screened and selected by phage display and genetically modified at the N-terminus of LAMP-2B to realize their targeting effects. Donor cells transfected with the plasmid produce exosomes displaying the engineered peptide ligands. For example, rabies virus glycoprotein (RVG) peptide (TIWMPENPRPGTPCDIFTNSRGKRASNG) shows selective binding to acetylcholine receptors and has been used to develop neuro-specific exosomes to deliver drugs to the central nervous system 47. Intravenously injected miRNA-124-loaded RVG exosomes were shown to penetrate ischemic regions of the cortex and promote neurogenesis 48. In another experiment, exosomes expressing the αγ integrin-specific iRGD peptide (CRGDKGPDC) fused to LAMP-2B efficiently delivered doxorubicin (DOX) to integrin-positive breast cancer cells in vitro and in vivo after intravenous injection 49. iRGD exosomes were also used to deliver KRAS siRNA specifically to αvβ3-harboring A549 tumors in vivo, resulting in specific *KRAS* gene knockdown and tumor growth suppression 50. tLyP-1 peptide (CGNKRTR), a non-small cell lung cancer (NSCLC)-homing peptide, selectively targets neuropilin-1 (NRP1) and neuropilin-2 (NRP2) receptors in the cell membrane. tLyp-1 exosomes selectively delivered siRNA to human NSCLC cells 51. In a recent report, we engineered chondrocyte affinity peptide (CAP, DWRVIIPPRPSA) exosomes that specifically delivered miRNA-140(miR-140)to chondrocytes in the joints and attenuated the progression of osteoarthritis (OA) in a rat model (**Figure 5**) 52. Exosomes displaying the mesenchymal stem cell (MSC) affinity peptide E7 have also been used to deliver a small molecule kartogenin (KGN) to synovial fluid-derived MSCs (SF-MSCs) to promote their chondrogenic differentiation and repair OA cartilage 53. Despite the effectiveness of this genetic method, the targeting peptide can be degraded in some situations. To enhance the stability of peptides displayed on the surface of exosomes, a glycosylation motif (GNSTM) can be added to the N-terminus of peptide-LAMP-2B fusions 54.

LAMP-2B can also be genetically fused with targeting proteins or antibody fragments to display antibodies on exosomes. Compared with peptides, which have modest binding affinities, antibodies or affibodies can achieve higher affinities, with *K*_D_ values in the low nanomolar range towards receptors on target cells. For example, to improve therapy of chronic myeloid leukemia (CML), researchers modified the surface of exosomes by fusing interleukin-3 (IL-3) to the N-terminal of LAMP-2B. IL-3 is the native ligand of interleukin-3 receptor α (IL-3Rα), which is highly expressed on CML blasts. IL-3 exosomes loaded with imatinib and BCR-ABL siRNA effectively killed CML cells and extended the overall survival rate of xenografted mice. In vivo tracking of these engineered exosomes showed rapid migration to the CML xenograft tumors 55. In another example, a HER2-binding affibody zHER was fused to the N-terminus of LAMP-2. zHER exosomes showed high binding affinity and selectivity for HCT-116 colon cancer cells and specifically delivered 5-FU and anti-miRNA-21 drugs to HER2-expressing tumors in vivo 56. Similarly, exosomes expressing HER2-binding ankyrin repeat protein (DARPin) delivered siRNA to HER2-positive human tumor cells 57. Wang et al*.* developed cardiac cell-targeted exosomes expressing ischemic myocardium-targeting peptide (IMPT, CSTSMLKAC) in MSCs. IMPT exosomes specifically localized to the ischemic myocardium area and exerted cardioprotective effects in acute myocardial infarction 58.

Besides LAMP-2B, the transmembrane protein platelet-derived growth factor receptor (PDGFR) is commonly used for membrane display. In one example, GE11 (YHWYGYTPQNVI) was genetically fused to the PDGFR transmembrane region using a pDisplay vector to produce GE11 exosomes in 293T cells. GE11 exosomes showed high affinity for epidermal growth factor receptor (EGFR)-overexpressing cancer cells and low mitogenic activity, making them a promising delivery system for EGFR-targeted therapy. In animals, GE11 exosomes loaded with the antitumor nucleic acid inhibitor miRNA let-7 significantly inhibited tumor growth, consistent with their tumor-targeting ability 59. In another example, two genes encoding scFv fragments of αEGFR cetuximab and αCD3 UCHT1 were inserted between the N-terminal signal peptide and the transmembrane domain of PDGFR. The cetuximab exosomes showed high sensitivity and specificity for EGFR-overexpressing cells, and the UCHT1 exosomes specifically recognized T-cells. These bispecific exosomes are promising platforms for cancer immunotherapy 60.

The tetraspanin superfamily CD63/CD9/CD81, with their two extracellular loops, can also be engineered to display targeting sequences or probes. For example, the fluorescent protein pHluorin was inserted into the small extracellular loop to probe for exosome secretion and uptake 61. In another example, ApoA-1, the main structural and functional protein of high-density lipoprotein (HDL), was displayed on the exosome membrane to bind scavenger receptor class B type 1 (SR-B1). SR-B1 is highly expressed on the surface of many types of hepatocellular carcinoma cells. ApoA-1 was genetically inserted into the small extracellular loop of CD63 and expressed as a fusion protein to facilitate its presentation on the surface of exosomes. Exosomes purified from ApoA-1-overexpressing donor cells functionally delivered miRNA-26a to SR-B1-expressing HepG2 cells, thus inhibiting hepatocarcinoma proliferation and migration in vitro 62. To develop a treatment for Duchenne muscular dystrophy (DMD), a common myopathic disease in children caused by loss of a functional dystrophin protein, the inhibitory domain of myostatin propeptide was fused with the second extracellular loop of CD63, which significantly improved its serum stability and delivery efficiency. Repeated injection of myostatin propeptide exosomes promoted muscle restoration and proliferation, resulting in notable blockade of muscle degradation and pathology in mdx mice 63.

Exosomes with surface-displayed antigens can also be used as anticancer vaccines. In one study, fusion of ovalbumin (OVA) antigen to CD63 produced OVA exosomes that improved the immunogenicity of DNA vaccines and prevented tumor growth in a xenograft model 64. Membrane-bound antigen cargo can also be displayed by the C1C2 domain of lactadherin, which binds and localizes to phosphatidylserine-containing exosome membranes, to increase levels of cytokines that support immunogenicity. Exosome targeting of tumor antigen vaccines successfully suppressed tumor growth in vivo 65. Further examples of fusion proteins based on lactadherin designed for exosome display include Gaussia luciferase-lactadherin and streptavidin-lactadherin 66-68.

Exosome membrane attachment via a signal peptide is also a readily achievable method for membrane display 69. For example, the C-terminus of a protein, such as an antibody, reporter protein, or nanobody, can be linked to the 37-residue glycosylphosphatidylinositol (GPI) signal peptide DAF, which can then be anchored into the exosome surface. In one study, GPI-anchored EGFR-specific nanobodies were utilized to specifically target EGFR-positive A431 cells 70. Other signal peptides have also been used. For example, a palmitoylation signal peptide (MLCCMRRTKQ) was genetically fused to the N-terminus of a fluorescent protein and anchored to the exosome membrane for membrane labeling 71. These surface-anchored chimera-bearing exosomes represent a feasible strategy for creating novel targeted therapeutics.

### Chemical modification

Although less explored, the surface of exosomes can be modified via chemical methods. Examples of chemically modified exosomes for targeted drug delivery are summarized in **Table 3**. For example, the amine groups of exosomal proteins can be easily modified with alkyne groups. Then, the alkyne-labeled exosomal proteins can be bio-orthogonally couple to azide-containing reagents through copper-catalyzed azide-alkyne cycloaddition (CuAAC) “click” reactions. In a proof-of-concept study, this approach was utilized to modify the exosome surface with both a small molecule dye and a larger azide-containing model protein 73. The glioma-targeting RGE peptide (RGERPPR) was conjugated to exosomes by a cycloaddition reaction with sulfonyl azide. After intravenous administration, RGE exosomes penetrated the BBB and targeted tumor regions. Targeted therapy using RGE exosomes loaded with curcumin had a strong anticancer effect in tumor-bearing mice 74. In another study, the peptide c(RGDyK) (Arg-Gly-Asp-D-Tyr-Lys) was conjugated to the exosome surface by a bioorthogonal click reaction. c(RGDyK) exosomes loaded with curcumin were found to efficiently penetrate the BBB and suppress inflammatory responses and cellular apoptosis in a transient middle cerebral artery occlusion mouse model 75. Chemical modifications can also be used to conjugate large biomolecules onto exosomes. Most tumors harbor surface CD47 that interacts with signal-regulating protein α (SIRPα) on phagocytes, which reduces the ability of macrophages to phagocytose tumor cells. Researchers designed an exosome-based immune checkpoint blocker that antagonizes the interaction between CD47 and SIRPα 76, 77. Azide-modified exosomes were clicked with dibenzocyclooctyne-derivatized SIRPα antibodies. The chemically modified exosomes were targeted to interfere with the CD47-SIRPα checkpoint on the surface of tumor cells, and their administration ameliorated engulfment of tumor cells by immune cells. Chemical modification of exosomes by click chemistry relies on conversion of exosomal amine groups to alkynes. This reaction, however, is not site specific as one cannot control which amino groups (e.g., N-terminal amino group, ε-amino groups of lysine residues) or which proteins are modified. Non-site-specific chemical modification might shield some protein-protein interactions and alter the recognition properties of the exosomes.

Insertion of amphipathic molecules into the lipid bilayer of exosomes represents another chemical modification strategy. Previous research has demonstrated that polyethylene glycol (PEG)-grafted 1,2-dioleoyl-*sn*-glycero-3-phosphoethanolamine (DSPE-PEG) can accumulate in the exosome membrane. This method was successfully used to immobilize DSPE-PEG-RGD on exosomes. When combined with the tumor-specific targeting ligand folate, nearly all injected RGD exosomes concentrated in tumor areas. Chemically modified RGD exosomes were also combined with photothermal therapy and chemotherapy for pro-angiogenic treatment. As sigma receptors are overexpressed in lung cancer, they have been proposed as receptors for targeted exosome delivery. Anisamides, including aminoethyl anisamide (AA), are high-affinity sigma-selective ligands. AA linked to DSPE-PEG was successfully conjugated to the exosome membrane 78. Compared with unmodified exosomes, AA-modified exosomes demonstrated enhanced uptake in lung cell lines, and targeted delivery of PTX enhanced therapeutic efficiency in vivo. These studies suggest that DSPE-PEG-based ligand-modified exosomes are promising drug carriers for tumor-targeted drug delivery. Since DSPE-PEG was approved by the FDA for medical applications, it has been extensively used to assemble targeting motifs on exosome membranes 79. Like DSPE, chol can self-assemble into exosomal membranes due to its hydrophobicity. In one study, exosomes were surface-modified with chol conjugated to RNA aptamers (PSMA-aptamer, EGFR-aptamer) or folate 80. These targeted exosomes delivered siRNA and miRNA to the corresponding tumor sites and enhanced antitumor efficacy. Another study explored the potential of chol conjugated to AS1411 aptamer to mediate the targeted delivery of exosomes to nucleolin-expressing tumor tissue 81. Other lipids have also been used to tether targeting ligands to the exosome surface. For example, a diacyl lipid-DNA aptamer (sgc8) conjugate was developed for cancer cell-specific therapy 82. In a study comparing the labeling efficiency of C18-PEG2000, DSPE-PEG2000 and chol-PEG2000, chol-conjugated exosomes were found to be optimal and provided outstanding stability at low temperature 83.

Paclitaxel (PTX)-loaded AS1411-chol exosomes were efficiently delivered to target cancer cells, providing a promising delivery platform for cancer therapy. Another study labelled exosomes with ApoA-1 mimetic peptides conjugated to lipids, which significantly enhanced their selective internalization by primary glioma cells 84. More interestingly, intravenously injected exosomes bypassed the BBB and were delivered into the glioma site as a brain cancer therapy. In another example, a membrane-targeted chimeric peptide (ChiP) was developed to anchor a nuclear localization signal (NLS) peptide to the exosomal membrane 85. Both in vitro and in vivo experiments demonstrated that ChiP exosomes facilitated nuclear delivery of photosensitizers and enhanced photodynamic tumor therapy 85. Chemical modification of the exosomal surface can be used not only for targeted drug delivery but also for targeted delivery of active exosomes. Display of a RVG peptide ligand linked to DOPE on the surface of MSC-derived exosomes greatly enhanced their binding to the cortex and hippocampus upon intravenous administration, which prevented memory deficits in an animal model of Alzheimer's disease 86.

### Membrane fusion

The lipid bilayer membrane of exosomes can spontaneously fuse with other types of membrane structures. In one study, fusogenic exosomes harboring the viral fusogen vascular stomatitis virus (VSV)‐G protein directly delivered membrane proteins into target cells, thus providing a new tool for membrane protein therapy 89. Fusion of exosomes and virus-simulating vesicles (Vir-FV) forms hybrid exosomes 90. Hybrid exosomes fused with liposomes have been used to deliver the CRISPR-Cas9 system for targeted gene editing 91, and to enhance the anti-tumor activity of cancer drugs 92. Tareste et al*.* used electrostatic interactions to induce fusion of cationic lipids with exosomes 93. The strong cationic charge enhanced exosome binding to the recipient cell and cellular uptake. Incubating synthetic azide-bearing liposomes with donor cells can also trigger the cells to secrete exosomes with azide groups on their lipid membranes. Subsequent bioconjugation with targeting peptides through controlled click reactions enhances the tumor-targeting ability of these exosomes. In one study, exosomes conjugated with CGKRK peptide were loaded with PTX for targeted delivery to B16F10 tumors 88.

### Summary

**Figure 6:**
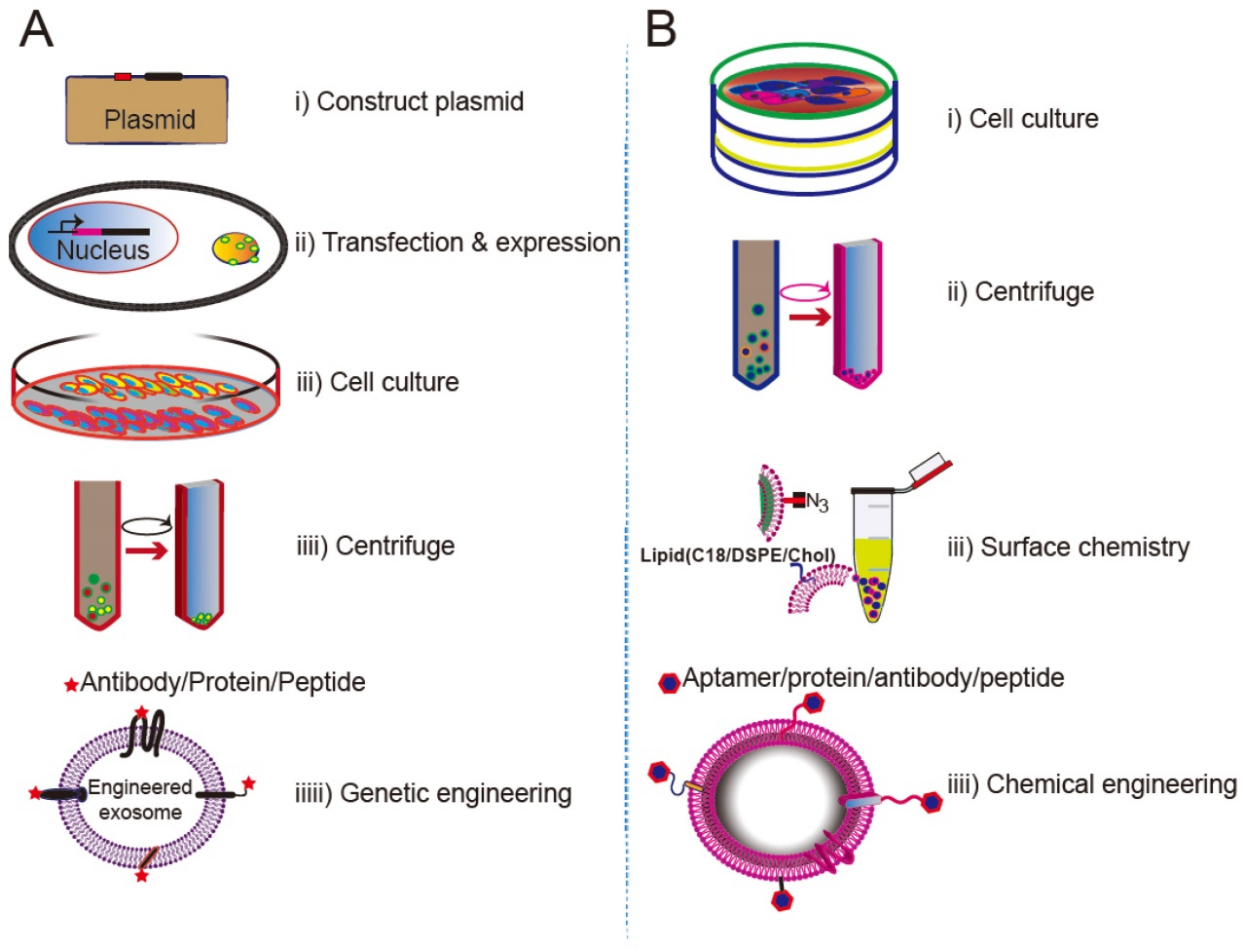
** Approaches for engineering exosomes displaying targeting ligands.** (A) Transfecting/infecting the donor cells with a plasmid encoding a fusion protein of the ligand and a selected membrane protein. (B) Surface modification via bioconjugation or lipid incorporation.

The procedures for creating cell-specific exosomes by genetic engineering or surface chemistry approaches are summarized in **Figure 6**. In genetic engineering approaches, a targeting ligand is genetically fused with an exosome membrane protein and subsequently overexpressed in the donor cells. The donor cells thereby produce genetically engineered exosomes that display the targeting ligand. Genetic engineering of exosomes represents a highly accessible strategy for the display of functional ligands on the exosomal membrane, but requires plasmid construction and overexpression of the proteins in the donor cells. Lipids or a bioconjugation reaction can also anchor a targeting moiety to the exosome membrane. Chemical methods rely on bioconjugation of the targeting ligand with surface proteins, but inactivation of the surface protein or aggregation of the exosome may occur during the chemical manipulation. Despite their limitations, both approaches have been successfully implemented.

## Perspective

Exosomes can transfer encapsulated proteins and genetic information to recipient cells and act as information messengers between cells. The surface molecules anchored on exosomes from different cell sources vary, which endows them with selectivity for specific recipient cells. Surface engineering aims to increase the local concentration of exosomes at the diseased site, thereby reducing toxicity and side effects and maximizing therapeutic efficacy. Recently, a series of transactions by large pharmaceutical companies have shown that the industry expects exosomes to deliver drugs to hard-to-reach tissues 94. Although surface engineering is widely used in targeted drug delivery, how it impacts the stability of exosomes, their cellular entry pathways, and tissue distribution in vivo still need to be elucidated.

Cell-targeting exosomes can be used for imaging studies or diagnosis. For example, bioluminescence and fluorescence tracking of exosomes were realized by enzymatic biotinylation of exosomes with biotin ligase 95. This application has been extensively reviewed elsewhere 23. Exosomes can also be combined with other biomaterials or inorganic materials for biomedical uses. Exosomes with metal-organic framework nanoparticles have shown increased drug-loading efficiencies and release properties and protection from enzyme-mediated protein degradation 96, 97. The combination of exosomes with metal-based nanoparticles also leads to enhanced radiotherapy and radiodynamic therapy. DNA nanostructures can also decorate the exosome surface, as exemplified by the in situ assembly of six-nucleotide aptamers upon molecular recognition 98. As early cancer diagnosis and specific tumor tracers are among the biggest challenges in cancer therapy, the rapidly developing exosome technologies provide powerful tools for integrating diagnosis and treatment. The future of exosome therapy includes combinations of targeted exosomes with anticancer drugs and high-precision cancer diagnostic probes to construct multifunctional platforms for in vivo tracking, prognosis monitoring, and therapy.

## Figures and Tables

**Table 1:** Summary of exosomal compositions. 36

	Composition Examples	
Proteins	Cytosolic proteins	clathrin, HSC70, HSP70, HSP60, HSP90, ALIX, YWHAE, ubiquitin, TSG101, ESCRT
	Cell surface proteins	CD63, CD9, CD81, CD37, CD68, CD82, LAMP-2B, MHCI, MHCII 37
	Membrane-associated proteins	annexins I, II, IV, V, VII; RAB7, RAB11, RAB1B
	Cytoskeletal proteins	tubulin, actin, cofilin, profilin I, elongation factor-1a, fibronectin
	Enzymes	ATPase, pyruvate kinase, fatty acid synthase 5
Lipids	sphingomyelins, phosphatidylserines, phosphatidylethanolamines, phosphatidylcholines, phosphatidylinositols, glycosphingolipid GM3, ceramides, fatty acids, glycerolipids, glycerophospholipids, sterol lipids, steroids 38, 39	
Nucleic acids	mRNAs, miRNAs, noncoding RNAs, mtDNAs 40	
Metabolites	carboxylic acids, amino acids, sugars, carnitines, biogenic amines, vitamins, cyclic alcohols 41	

**Table 2:** Genetically engineered exosomes as drug delivery systems.

Therapeutic cargo	Targeting ligand	Method	Target cells	Function	Reference
BACE1 siRNA	RVG peptide	LAMP-2B	neuronal cells (Neuro-2a)	Alzheimer's disease therapy	35
miRNA-124	RVG peptide	LAMP-2B	cortical neural progenitors	Promote neurogenesis after ischemia	35
KRAS siRNA	iRGD peptide	LAMP-2B	adenocarcinoma, human alveolar basal epithelial cells (A549)	Target oncogenic KRAS	49
DOX	iRGD peptide	LAMP-2B	breast cancer	Targeted delivery of DOX	49
SOX2 siRNA	Tlyp-1	LAMP-2B	non-small cell lung cancer, A549 stem cells	Gene delivery for cancer therapy	51
miRNA-140	CAP peptide	LAMP-2B	chondrocytes	Attenuate the progression of osteoarthritis	52
KGN	E7 peptide	LAMP-2B	SF-MSCs	Cartilage regeneration	53
imatinib, BCR-ABL siRNA	IL-3	LAMP-2B	chronic myelogenous leukemia cells (LAMA84, K562, K562R)	Inhibit cancer cell growth	55
5-fluorouracil anti-miRNA-21	zHER affibody	LAMP-2B	colorectal cancer (HCT-116)	Reverse chemoresistance and improve cancer treatment efficiency	56
Tpd50 siRNA	DARPin	LAMP-2B	HER2-positive cells (SKBR3)	RNAi therapy of HER2-positive cancer	57
MSC exosomes	IMTP peptide	LAMP-2B	cardiomyocytes (H9c2)	Repair after myocardial infarction	58
miRNA-let7a	GE11 peptide	LAMP-2B	breast cancer (HCC70)	Target EGFR-expressing tumor	59
Smart-exos	αCD3/αEGFR	Smart-exos	T-cells (Jurkat), EGFR-positive breast cancer (MDA-MB-468)	Cell-free cancer immunotherapy	60
miRNA-26a	ApoA-1	CD63	hepatocellular carcinoma (HepG2)	Suppress tumor cell migration and proliferation	62
antigen	OVA antigen	CD63	CD8+ T-cells	Improve the immunogenicity of cancer vaccines	64-65
MSC exosomes	CMP peptide	LAMP-2B	cardiomyocytes	Targeted delivery of MSC exosomes	72

**Table 3:** Chemically modified exosomes for targeted drug delivery.

Therapeutic cargo	Targeting ligand	Method	Target cells	Function	Reference
curcumin-SPION	neuropillin-1-targeted peptide	click chemistry	glioma (U251)	Simultaneous diagnosis and treatment of glioma	74
curcumin	c(RGDyk)	click chemistry	cerebral vascular endothelial cells (integrin αvβ3-positive U87)	Reduce inflammation and inhibit apoptosis in ischemic stroke	75
PTX	AA	DSPE-PEG-AA	murine lung cancer (3LL-M27), sigma receptor-positive cells	Improve drug circulation and inhibit pulmonary metastases	78
quantum dot photothermal agent	RGD	DSPE-PEG-RGD	breast cancer (integrin αvβ3-poitive MCF-7)	Photothermal therapy	79
erastin	folate	DSPE-PEG-folate	breast cancer (MDA-MB-231)	Targeted induction of ferroptosis	80
surviving siRNA	PSMA RNA aptamer, EGFR RNA aptamer, folate	chol	breast cancer (MDA-MB-468), prostate cancer, colorectal cancer	Tumor-targeted RNAi nanomedicine	80
miRNA-let7, VEGF siRNA	AS1411 aptamer	chol	nucleolin-positive cancer cells (MDA-MA-231)	Tumor-targeted small RNA delivery	80
DOX	sgc8 aptamer	diacyl lipid-(PEG)2	leukemia cells	Targeted anti-cancer therapy	82
PTX	AS1411	chol-PEG2000	breast cancer (MDA-MA-231)	Targeted anti-cancer chemotherapy	83
methotrexate, KLA (Lys-Leu-Ala)	ApoA-1 mimetic peptide	lipid	glioma	Selective brain tumor treatment	84
photosensitizer	NLS peptide	C16	carcinoma (4T1), colorectal cancer (CT26)	Nuclear-targeted and enhanced photodynamic therapy	85
aSIRPα, aCD47	antibodies	azide‐modified	macrophages and tumor cells	Enhance phagocytosis of cancer cells by blocking SIRPα-CD47 interaction	76
mannosamine	RGD	DSPE-PEG-RGD	αvβ3 overexpressing cells (HUVEC)	Promote angiogenesis with targeted imaging	87
PTX, tirapazamine	D-CGKRK	CuAAC	B16F10	Hybrid membrane vesicles for targeted therapy	88
